# Combined whole exome sequencing and chromosomal microarray analysis improve clinical interpretation of genomic variants in patients with intellectual disability

**DOI:** 10.1192/j.eurpsy.2023.1878

**Published:** 2023-07-19

**Authors:** A. A. Kashevarova, E. O. Belyaeva, E. A. Fonova, M. E. Lopatkina, O. Y. Vasilyeva, D. A. Fedotov, A. A. Zarubin, A. A. Sivtsev, V. V. Demeneva, O. A. Salyukova, V. V. Petrova, S. V. Fadiushina, L. I. Minaycheva, G. N. Seitova, L. P. Nazarenko, I. N. Lebedev

**Affiliations:** 1 Research Institute of Medical Genetics, Tomsk National Research Medical Center, Russian Academy of Sciences, Tomsk, Russian Federation

## Abstract

**Introduction:**

aCGH determines pathogenic copy number variations (CNVs) in about 10% of patients with intellectual disability (ID). In another 20% of patients, probably pathogenic CNVs or variants with uncertain clinical significance are detected. It may be variants that do not fully explain the patient’s symptoms, aberrations with reduced penetrance or inherited from healthy parents. The use of a sequencing method for such cases is advisable.

**Objectives:**

Improvement of diagnosis of intellectual disability.

**Methods:**

aCGH with 60K Agilent microarrays, qPCR, targeted sequencing, whole exome sequencing (WES).

**Results:**

Six patients with ID and inherited deletions/duplications detected by aCGH and their parents if available were further examined by sequencing. Four patients had maternal CNVs: (1) del1q41 (*SPATA17, LINC00210, RRP15*), (2) del7q35 (*TCAF2*, exon 8), (3) dup8p22p21.3 (*PSD3,* exons 1-11), and (4) del12p11.1 (*SYT10,* exons 1-2). Two patients had paternal CNVs: (5) dup1q44 (*SMYD3*, exons 2-5) and (6) del15q11.2 (*TUBGCP5, CYFIP1, NIPA1, NIPA2, LOC283683*). The severe phenotype of patient (5) with dup1q44 could not be explained by the paternally inherited disruption of the single *SMYD3* gene. WES determined probably pathogenic SNV in the *MID1* gene associated with Opitz GBBB syndrome (OMIM 300000), which corresponds better to the patient’s phenotype and is likely to be the cause of the disease. Although del1q41 is included in the region of chromosome 1q41-q42 deletion syndrome (OMIM 612530) the phenotype of the patient (1) is much milder; WES in the patient detected two pathogenic (*MPO, MAN2C1*) and one probably pathogenic (*ARID1B*) SNVs. In patient (6) with del15q11.2 pat WES detected additional pathogenic SNV in exon 7 of the *ARSE* gene. In patient (3) with dup8p22p21.3 WES determined two SNVs with uncertain significance in the *KIDINS220, FOXG1* genes. No SNVs were detected by WES in patient (2) with del7q35. For patient (4) with del12p11.1 targeted *SYT10* sequencing revealed no pathogenic SNVs as well.

**Conclusions:**

Sometimes aCGH-analysis is sufficient to identify the causes of ID, however, in the case of detection of CNVs with uncertain clinical significance and/or inherited from healthy parents, it may be necessary to further examine the patient using sequencing methods. So, the accurate diagnosis was made by WES for one patient of eight. For another two patients the combination of CNVs and SNPs should be considered. For the last three patients the described aberrations could not explain the phenotype and whole genome sequencing may be the solution.This study was supported by the Russian Science Foundation, grant 21-65-00017, https://rscf.ru/project/21-65-00017/

**Disclosure of Interest:**

None Declared

